# Influence of the *FCGR2A* rs1801274 and *FCGR3A* rs396991 Polymorphisms on Response to Abatacept in Patients with Rheumatoid Arthritis

**DOI:** 10.3390/jpm11060573

**Published:** 2021-06-18

**Authors:** Noelia Márquez Pete, María del Mar Maldonado Montoro, Cristina Pérez Ramírez, Fernando Martínez Martínez, Juan Enrique Martínez de la Plata, Abdelali Daddaoua, Alberto Jiménez Morales

**Affiliations:** 1 Pharmacy Service, Pharmacogenetics Unit, University Hospital Virgen de las Nieves, 18014 Granada, Spain; noeliamarquezpete@gmail.com (N.M.P.); alberto.jimenez.morales.sspa@juntadeandalucia.es (A.J.M.); 2 Clinical Analysis Service, Hospital Campus de la Salud, 18016 Granada, Spain; mariadelmarmaldonadomontoro@gmail.com; 3 Center of Biomedical Research, Department of Biochemistry and Molecular Biology II, Institute of Nutrition and Food Technology “José Mataix”, University of Granada, Avda. del Conocimiento s/n., 18016 Granada, Spain; 4 Department of Pharmacy and Pharmaceutical Technology, Social and Legal Assistance Pharmacy Section, Faculty of Pharmacy, University of Granada, 18071 Granada, Spain; femartin@ugr.es; 5 Pharmacy Service, Hospital de Poniente-El Ejido, 04700 El Ejido, Spain; juanenriquemartinezdelaplata@gmail.com; 6 Department of Biochemistry, Faculty of Pharmacy, University of Granada, 18071 Granada, Spain; daddaoua@ugr.es

**Keywords:** rheumatoid arthritis, abatacept, *FCGR2A*, FCGR3A, effectiveness, polymorphisms

## Abstract

Abatacept (ABA) is an immunosuppressant indicated for treatment of rheumatoid arthritis (RA). Effectiveness might be influenced by clinical RA variants and single-nucleotide polymorphisms (SNPs) in genes encoding protein FCGR2A (His131Arg) and FCGR3A (Phe158Val) involved in pharmacokinetics of ABA. An observational cohort study was conducted in 120 RA Caucasian patients treated with ABA for 6 and 12 months. Patients with the *FCGR2A* rs1801274-AA genotype (FCGR2A-p.131His) showed a better EULAR response (OR = 2.43; 95% CI = 1.01–5.92) at 12 months and low disease activity (LDA) at 6 months (OR = 3.16; 95% CI = 1.19–8.66) and 12 months (OR = 6.62; 95% CI = 1.25–46.89) of treatment with ABA. A tendency was observed towards an association between the *FCGR3A* rs396991-A allele (FCGR3A-p.158Phe) and better therapeutic response to ABA after 12 months of treatment (*p* = 0.078). Moreover, we found a significant association between the low-affinity *FCGR2A/FCGR3A* haplotypes variable and LDA after 12 months of ABA treatment (OR = 1.59; 95% CI = 1.01–2.58). The clinical variables associated with better response to ABA were lower age at starting ABA (OR = 1.06; 95% CI = 1.02–1.11) and greater duration of ABA treatment (OR = 1.02; 95% CI = 1.01–1.04), lower duration of previous biological therapies (OR = 0.99; 95% CI = 0.98–0.99), non-administration of concomitant disease-modifying antirheumatic drugs (DMARDs) (OR = 24.53; 95% CI = 3.46–523.80), non-use of concomitant glucocorticoids (OR = 0.12; 95% CI = 0.02–0.47), monotherapy (OR = 19.22; 95% CI = 2.05–343.00), lower initial patient’s visual analogue scale (PVAS) value (OR = 0.95; 95% CI = 0.92–0.97), and lower baseline ESR (OR = 0.92; 95% CI = 0.87–0.97). This study showed that high-affinity FCGR2A-p.131His variant, low-affinity FCGR3A-p.158Phe variant, and combined use of *FCGR2A/FCGR3A* genetic variations could affect ABA effectiveness. Further studies will be required to confirm these results.

## 1. Introduction

Abatacept (ABA) is an immunosuppressant indicated for treatment of rheumatoid arthritis (RA), psoriatic arthritis, and juvenile idiopathic arthritis^1^. ABA comprises a fusion of the extracellular domain of human cytotoxic T-lymphocyte-associated protein 4 (CTLA4) with the fragment crystallizable (Fc) region of human immunoglobulin G1 (IgG1) [1]. The mechanism of action of ABA is based on interaction of CTLA4 with the CD80/CD86 complex, preventing the latter from binding with the CD28 membrane receptor of T lymphocytes [2]. Consequently, the co-stimulatory signal for T cell activation is blocked [2]. The function of the Fc region of IgG is to improve the pharmacokinetics of ABA, increasing its stability and prolonging the half-life of the drug [3,4]. In addition, the Fc region of IgG1 binds to the Fc-gamma receptors (FCGRs) given rise to a series of immune reactions such as apoptosis, cytokine release, antibody-dependent cellular cytotoxicity (ADCC), and macrophage-mediated immune complex elimination [5,6,7,8]. For this reason, the FCGRs play a part in innate and acquired immune activation and have been extensively investigated for their role in the pharmacogenetic of biological therapies (BTs) [4,9,10,11,12]. Several subtypes of FCGRs have been described, the most extensively studied being FCGR2A and FCGR3A [13,14]. The protein FCGR2A is expressed on monocytes, macrophages, dendritic cells, neutrophils, and platelets, inducing immune reactions such as phagocytosis of opsonized IgGs, ADCC, reactive oxygen species production, and cytokine production [15]. Similarly, FCGR3A is expressed on monocytes, macrophages, neutrophils, and NK cells, promoting phagocytosis and ADCC mechanisms [15]. The affinity of FCGR2A and FCGR3A for the Fc region of IgG of ABA could vary due to structural changes in extracellular domain of the receptors, which could interfere with the therapeutic response to ABA [13,14]. The FCGR structural changes could be produced by single-nucleotide polymorphisms (SNPs) located in the genetic coding region of *FCGR2A* and *FCGR3A* [13,14]. The *FCGR2A* rs1801274 (A > G) polymorphism gives rise to a histidine (His) to arginine (Arg) substitution (His131Arg) [16,17]. As has been observed in previous studies, the FCGR2A with His instead of Arg at position 131 showed greater affinity for IgG1 [18]. The rs396991 (A > C) polymorphism located on the *FCGR3A* gene produces a phenylalanine (Phe) to valine (Val) substitution (Phe158Val) [19]. The Val158 variant showed a greater affinity for IgG1, IgG2, and IgG3, which was associated with greater immune response (ADCC, apoptosis) [19,20]. Studies conducted on tumor necrosis factor inhibitors (TNFis) have evaluated the relationship of these SNPs to the effectiveness and variation in clearance of the drug, obtaining contradictory results [4,9,11,21,22,23,24,25]. The low affinity variants FCGR2A-p.131Arg and FCGR3A-p.158Phe could decrease the binding of ABA to the receptors, and thus its clearance would be lower, increasing the therapeutic effect [18]. Cañete et al. showed that patients undergoing treatment with infliximab (IFX) and carrying the *FCGR2A* rs1801274-GG and *FCGR3A* rs396991-AA genotypes, both low-affinity, exhibited lower clearance of the drug, and that its therapeutic effect, and consequently the response to therapy, therefore increased [18]. Similarly, these SNPs are related to the development of autoimmune diseases [26]. Low-affinity receptors could present less binding and clearance of autoimmune complexes, increasing tissue damage in patients carrying low-affinity variants and producing less response to treatment [15]. Another recent study, performed by Jiménez Morales et al., identified the high-affinity genotype *FCGR2A* rs1801274-TT and allele *FCGR3A* rs396991-G as predictors of greater therapeutic response to rituximab (RTX) and the low-affinity genotype *FCGR3A* rs396991-TT as a predictor of good response to tocilizumab (TCZ) [9]. Theories about the influence of *FCGR2A* and *FCGR3A* SNPs in response to BTs are multiple and the effect of the interaction of these receptors with biologic drugs is unknown. Nevertheless, these SNPs could determine the great interindividual variability in the response to ABA; the therapeutic failure rate is approximately 30% [27]. However, no previous study has evaluated the relationship between the FCGR2A rs1801274 and FCGR3A rs396991 SNPs and ABA response in patients diagnosed with RA. Recently, SNPs have been investigated in genes related to the mechanism of action of ABA, such as *CTLA-4* (rs5742909, rs231775), which could be associated with ABA response [28].

Within this conceptual framework, the objective of this study was to evaluate the involvement of the *FCGR2A* rs1801274 and *FCGR3A* rs396991 SNPs and low-affinity *FCGR2A/FCGR3A* haplotypes as possible biomarkers of response to ABA (EULAR response, low disease activity (LDA), and remission) after 6 and 12 months of treatment in patients diagnosed with RA.

## 2. Materials and Methods

### 2.1. Study Design

We conducted a retrospective observational cohort study.

### 2.2. Ethics Statements

The study was carried out with the approval of the Ethics and Research Committee of the University Hospital Virgen de las Nieves (HUVN) in accordance with the Declaration of Helsinki. The subjects who participated in the study signed an informed consent for collection and genetic analysis of saliva samples and for their donation to the Andalusian Public Health System Biobank. The samples were identified by alphanumeric codes.

### 2.3. Study Population

The study was conducted at the HUVN in Granada, Spain. We recruited 120 patients over the age of 18 years diagnosed with RA in the Rheumatology Department of the HUVN, according to the American College of Rheumatology (ACR) classification criteria [29], treated with ABA for a period of at least 6 months. ABA was administered intravenously (IV), in doses of 500 mg (<60 kg), 750 mg (60–100 kg), or 1.000 mg (>100 kg), in weeks 0, 2, and 4, respectively, and every 4 weeks subsequently at the same doses in 52 patients, or subcutaneously (SC) at 125 mg/week in 68 patients.

### 2.4. Sociodemographic and Clinical Variables

The sociodemographic and clinical data were collected by reviewing clinical histories. The sociodemographic data collected were sex, smoking, age at RA diagnosis, number of years with disease, age at start and duration of ABA treatment, mode of administration of the drug (IV or SC), concomitant GCs, concomitant csDMARDs (methotrexate (MTX), leflunomide (LFN)), number and duration of previous BTs, and reason for suspension of ABA. In addition, the clinical data collected were Disease Activity Score in 28 joints (DAS28) [30,31,32], Health Assessment Questionnaire (HAQ) score, C-reactive protein (CRP) level, erythrocyte sedimentation rate (ESR), presence of rheumatoid factor (RF), anti-citrullinated protein antibodies (ACPA), number of painful joints (NPJ), number of swollen joints (NSJ), and patient visual analogue scale (PVAS).

### 2.5. Genetic Variables

#### 2.5.1. DNA Isolation

Following the patients’ inclusion and signing of the informed consent, saliva samples were collected with buccal swabs (OCR-100). The DNA was extracted using the QlAamp DNA Mini Kit (Qiagen GmbH, Hilden, Germany), following the manufacturer’s instructions for purifying DNA from saliva, and stored at −40 °C. The DNA concentration and purity were measured using a NanoDrop 2000 UV spectrophotometer with the absorbance ratio at 280/260 and 280/230.

#### 2.5.2. Detection of Gene Polymorphisms

The *FCGR2A* rs1801274 and *FCGR3A* rs396991 gene polymorphisms were analyzed by real-time polymerase chain reaction (PCR) using TaqMan^®^ probes (ABI Applied Biosystems, Waltham, MA, USA, 7300 Real-Time PCR System). The assay IDs used were *FCGR2A* (rs1801274) C___9077561_20 and *FCGR3A* (rs396991) C__25815666_10. The genetic variables were determined with StepOne v2.3 software.

### 2.6. Response Variables

The clinical response was evaluated after 6 and 12 months of ABA treatment and was categorized as EULAR response, LDA, or remission. The EULAR response was evaluated according to the European League Against Rheumatism criteria [33] and was classified as satisfactory (DAS28 < 3.2) or unsatisfactory (DAS28 ≥ 3.2) [29,34]. Low disease activity was established for values in the range 2.6 ≤ DAS28 ≤ 3.2 [29,32] and remission for DAS28 < 2.6 [29,33,35].

### 2.7. Statistical Analysis

The descriptive analysis was performed using R 3.5.1 software. The quantitative variables were expressed as the mean (±standard deviation) for those that complied with normality and as the median and percentiles (25 and 75) for the variables that did not follow a normal distribution. Normality was confirmed by the Shapiro–Wilk test. The bivariate analysis between the response and the genetic variables was performed using Pearson’s chi-squared test or applying Fisher’s exact test for the qualitative variables. For the quantitative variables, Student’s *t*-test was applied to the variables that complied with normality. The Mann–Whitney *U* test was applied for non-normal variables. The low-affinity *FCGR2A*/*FCGR3A* haplotype variable was analyzed as a quantitative variable. The alleles considered as low-affinity for the polymorphisms studied were *FCGR2A* rs1801274-G (FCGR2A-p.131Arg) and *FCGR3A* rs396991-A (FCGR3A-p.158Phe), whereas *FCGR2A* rs1801274-A (FCGR2A-p.131His) and *FCGR3A* rs396991-C (FCGR3A-p.158Val) are high-affinity alleles [18,19,20]. Transformation from a categorical to a quantitative variable was performed by assigning a score to each allele. A value of 0 was assigned to each of the *FCGR2A* rs1801274-A and *FCGR3A* rs396991-C alleles, being high-affinity alleles, while the value 1 was assigned to the low-affinity alleles *FCGR2A* rs1801274-G and *FCGR3A* rs396991-A. As each patient had four alleles, two for each polymorphism, the score assigned was from 0 to 4, where 0 signified the absence of low-affinity alleles (AACC) and 4 signified the presence of four low-affinity alleles (GGAA).

Multivariate analysis (logistic or linear regression) was used to calculate the adjusted odds ratio (OR) and 95% confidence interval (95% CI) for potential prognostic factors for EULAR response, LDA, and remission. The goodness of fit for each model was analyzed with the Hosmer–Lemeshow test and the omnibus test of coefficients, and the Cox–Snell and Nagelkerke *r^2^* coefficients were also calculated. All tests were two-sided, with a probability of 0.05 or less being considered statistically significant, and were performed using R 3.5.1 or PLINK toolset free-access software for whole-genome association analysis [36,37,38].

We determined the Hardy–Weinberg equilibrium and the haplotype frequencies and calculated Lewontin’s D-prime (D′) and the linkage disequilibrium coefficient (*r^2^*). The linkage disequilibrium (LD) for each polymorphism was calculated with the PLINK genome association analysis program [37]. The haplotype frequencies and their association with the response variable were analyzed using the snpStats program, a web-based tool for analysis of association studies [39,40,41,42,43].

## 3. Results

### 3.1. Patient Characteristics

A total of 120 patients receiving ABA as treatment were included in the study. The clinical and sociodemographic data are shown in Table 1. Of all the patients diagnosed with RA, the mean age at diagnosis was 45.15 ± 13.72 years; 74.17% (89/120) were women. All the patients were treated with other DMARDs for a median period of 36 (24–72) months. The median number of previous BTs was 2 (1–3). The percentage of ABA-naïve patients was 12.50% (15/120). The mean age at starting ABA was 56.63 ± 13.03 years, and the median duration of treatment with ABA was 24 (14.75–44.25) months. In our cohort, 5.83% (7/120) received ABA treatment as monotherapy during the study period. Concomitant MTX and GC were received by 35% (42/120) and 85% (102/120), respectively. The mean DAS28 score on starting ABA was 4.70 ± 1.43. The description of the clinical and sociodemographic parameters is shown in Table 1.

### 3.2. Clinical Effectiveness of ABA

The effectiveness of ABA was evaluated in 120 (93.75%) and 105 (82.03%) patients after 6 and 12 months of treatment, respectively (Table 2). After 6 months of ABA therapy, 31.67% (38/120) of individuals showed satisfactory EULAR response, 20% (24/120) had LDA, and 15% (18/120) had entered the remission phase of the disease. Furthermore, after 12 months of therapy with ABA, 45.71% (48/105) of subjects showed satisfactory EULAR response, 22.86% (24/105) exhibited LDA, and 27.62% (29/105) had remission of RA. In ABA-bionaïve patients, EULAR response was satisfactory in 53.33% (8/15) of cases after 6 months of treatment and increased to 78.57% (11/14) after 12 months of treatment. The percentage with LDA was 33.33% (5/15) after 6 months of treatment and 21.43% (3/14) after 12 months of treatment with ABA, while 20% (3/15) of patients attained remission after 6 months of ABA treatment, rising to 64.29% (9/14) after 12 months with ABA. All these results are set out in detail in Table 2.

### 3.3. Distribution of the Genotypes Analyzed

All the gene polymorphism distributions were in agreement with those expected according to the Hardy–Weinberg equilibrium (HWE) model (Table S1). The D′ linkage disequilibrium (LD) and *r^2^* values are given in Table S2. All the polymorphisms showed minor allele frequencies higher than 1%, and none of them were excluded from the analysis (Table S3).

### 3.4. ABA Response Predictors at 6 Months

#### 3.4.1. EULAR Response

After the bivariate analysis, greater EULAR response was found in patients with lower disease duration (OR = 0.94; 95% CI = 0.89–0.99), without concomitant GCs (OR = 4.30; 95% CI = 1.36–14.55), in monotherapy (OR = 14.82; 95% CI = 1.69–704.16), and with lower duration of treatment with previous BTs (OR = 0.98; 95% CI = 0.97–0.99) (Table S10). The clinical variables that showed an association with satisfactory EULAR response were lower baseline levels of DAS28 (OR = 0.42; 95% CI = 0.90–1.89), NPJ (OR = 0.84; 95% CI = 0.75–0.92), NSJ (OR = 0.79; 95% CI = 0.67–0.91), PVAS (OR = 0.94; 95% CI = 0.92–0.97), ESR (OR = 0.97; 95% CI = 0.94–0.99), and HAQ (OR = 0.32; 95% CI = 0.16–0.58) (Table S10).

With regard to the pharmacogenetic variables, a tendency was found in our patients towards association between the *FCGR2A* rs1801274-AA genotype and satisfactory EULAR response (AA vs. G; *p* = 0.056; Table S10). The multivariate analysis showed that the independent variables associated with satisfactory EULAR response after 6 months of treatment were lower duration of previous BTs (OR = 0.98; 95% CI = 0.97–0.99), non-administration of concomitant DMARDs (OR = 24.53; 95% CI = 3.46–523.80), and the *FCGR2A* rs1801274-AA genotype (AA vs. G; OR = 2.43; 95% CI = 1.01–5.92) (Table 3).

#### 3.4.2. Low Disease Activity (LDA)

In the bivariate analysis, it was found that lower values of the DAS28 (OR = 0.67; 95% CI = 0.47–0.93), initial PVAS (OR = 0.96; 95% CI = 0.94–0.98), and initial HAQ (OR = 0.49; 95% CI = 0.25–0.94) clinical variables were associated with LDA (Table S11). The *FCGR2A* rs1801274-AA genotype was found to be associated with LDA (AA vs. G; OR = 2.67; 95% CI = 0.96–7.44). In the multivariate analysis, LDA was found to be associated with a lower initial PVAS value (OR = 0.97; 95% CI = 0.95–0.99) and the *FCGR2A* rs1801274-AA genotype (AA vs. G; OR = 3.16; 95% CI = 1.19–8.66) (Table 3).

#### 3.4.3. Remission

Remission of the disease after 6 months of ABA treatment was associated, in the bivariate analysis, with patients who had been receiving ABA therapy for longer (OR = 1.02; 95% CI = 1.01–1.03), without concomitant GCs (OR = 5.16; 95% CI = 1.40–18.58), with ABA in monotherapy (OR = 9.13; 95% CI = 1.39–68.97) and with lower duration of previous BTs (OR = 0.98; 95% CI = 0.96–0.99) (Table S12). As for the clinical variables, remission was associated with lower values for initial DAS28 (OR = 0.44; 95% CI = 0.27–0.66), baseline NPJ (OR = 0.79; 95% CI = 0.66–0.91), baseline NSJ (OR = 0.68; 95% CI = 0.49–0.86), initial PVAS (OR = 0.95; 95% CI = 0.92–0.97), baseline ESR (OR = 0.94; 95% CI = 0.89–0.98), and initial HAQ (OR = 0.32; 95% CI = 0.14–0.67) (Table S12). The multivariate analysis showed that the independent variables associated with remission of the disease were greater duration of ABA therapy (OR = 1.02; 95% CI = 1.01–1.04), lower duration of previous BTs (OR = 0.98; 95% CI = 0.95–0.99), lower baseline ESR (OR = 0.92; 95% CI = 0.87–0.97), and monotherapy (OR = 19.22; 95% CI = 2.05–343.00) (Table 3).

### 3.5. ABA Response Predictors at 12 Months

#### 3.5.1. EULAR Response

In the bivariate analysis, a greater EULAR response was found in patients without concomitant GCs (OR = 4.42; 95% CI = 1.32–14.78) who were treated in monotherapy (OR = 7.86; 95% CI = 0.93–68.99) and were ABA-bionaïve (OR = 0.17; 95% CI = 0.03–0.71) (Table S10). As regards the clinical variables, a satisfactory EULAR response was found to be associated with lower values for initial DAS28 (OR = 0.68; 95% CI = 0.49–0.91), baseline NPJ (OR = 0.94; 95% CI = 0.87–1.01), initial PVAS (OR = 0.95; 95% CI = 0.93–0.97), and initial HAQ (OR = 0.39; 95% CI = 0.21–0.71) (Table S10). With respect to the pharmacogenetic variables, a tendency was found towards an association between the *FCGR3A* rs396991-A allele and satisfactory EULAR response (A vs. CC; *p* = 0.078) (Table S10). After the multivariate analysis, the independent variables associated with satisfactory EULAR response were a lower initial PVAS value (OR = 0.95; 95% CI = 0.92–0.97) and lower duration of previous BTs (OR = 0.99; 95% CI = 0.98–0.99) (Table 3).

#### 3.5.2. Low Disease Activity (LDA)

In the bivariate analysis, low disease activity was associated with individuals who started ABA therapy at an older age (OR = 1.04; 95% CI = 1.00–1.09) and who received it without concomitant GCs (OR = 3.29; 95% CI = 1.07-10.10) and in monotherapy (OR = 5.2; 95% CI = 1.08–25.30) (Table S11). An association was found between the *FCGR2A* rs1801274-G genotype and LDA (G vs. AA; OR = 4.57; 95% CI = 1.26–16.59) (Table S11). The multivariate analysis showed an association between LDA and older age on starting ABA (OR = 1.06; 95% CI = 1.02–1.11), non-use of concomitant GCs (OR = 0.12; 95% CI = 0.02–0.47), and the *FCGR2A* rs1801274-AG genotype (AG vs. AA/GG; OR = 12.82; 95% CI = 2.95–83.04) (Table 3).

#### 3.5.3. Remission

In the bivariate analysis, the variables associated with remission were lower number (OR = 0.64, 95% CI = 0.42–0.94) and lower duration (OR = 0.98; 95% CI = 0.97–0.99) of previous BTs (Table S12). Moreover, ABA-bionaïve patients showed greater remission (OR = 6.60; 95% CI = 1.67–29.62) (Table S12). The clinical variables associated with remission were lower values for baseline NPJ (OR = 0.91; 95% CI = 0.82–1.00), initial PVAS (OR = 0.97; 95% CI = 0.95–0.99), and initial HAQ (OR = 0.43; 95% CI = 0.22–0.80) (Table S12). In the multivariate analysis, the independent variables associated with remission of the disease were lower duration of previous BTs (OR = 0.98; 95% CI = 0.97–0.99) and a lower initial PVAS value (OR = 0.96; 95% CI = 0.94–0.98) (Table 3).

### 3.6. Association between Low-Affinity FCGR2A/FCGR3A Haplotypes and ABA Response

Significant association was found in the bivariate analysis between the low-affinity *FCGR2A/FCGR3A* haplotype variable and LDA after 12 months of treatment with ABA (OR = 1.59; 95% CI = 1.01–2.58) (Table S11). Moreover, a tendency was found towards an association between the low-affinity *FCGR2A/FCGR3A* haplotype variable and satisfactory EULAR response at 12 months of ABA therapy (*p* = 0.088) (Table S10).

Global haplotype analysis adjusted by sex, duration of previous BTs, DAS28, and monotherapy revealed that the AC (OR = 1.00), GA (OR = 1.90; 95% CI = 0.66–5.49), AA (OR = 0.90; 95% CI = 0.28–2.91), and GC (OR = 5.24; 95% CI = 0.98–28.08) haplotypes were associated with a higher EULAR response at 6 months of ABA therapy (*p* = 0.013) (Table 4). Haplotype frequency estimation values are given in Tables S4–S9.

## 4. Discussion

The interindividual response of patients diagnosed with RA and treated with BTs is very variable [33,44,45]. The search for biomarkers of response to these treatments is the main objective of numerous research studies carried out in recent years [9,12,46]. For this purpose, we need to evaluate the effectiveness of the treatments in different populations and find the biomarkers that determine that effectiveness. In our study, bionaïve patients showed a greater EULAR response after 6 and 12 months of ABA treatment (53.33% and 78.57%, respectively) compared to non-bionaïve patients, who had a lesser EULAR response to treatment with ABA at 6 and 12 months (31.67% and 45.71%, respectively). Furthermore, remission of the disease was greater in the bionaïve group than in the non-bionaïve group (64.29% vs. 27.62%) after 12 months of ABA treatment. A study conducted by Cagnotto et al. in 2716 Caucasian patients (from Sweden) with RA found a greater EULAR response in bionaïve patients after 12 months of treatment with ABA (OR_adjusted_ = 4.29, 95% CI = 2.77–6.65) [47]. Moreover, higher values in duration and number of previous BTs were identified as being responsible for a lower response to ABA treatment. The duration of the disease, and also age on starting treatment with ABA and duration of that treatment, have been identified in our study as predictors of ABA treatment response. Other studies have obtained the same results, highlighting the importance of early treatment of RA to achieve remission or, failing that, LDA [48,49,50]. In our patients, the administration of ABA in monotherapy, without concomitant GCs, was associated with a satisfactory EULAR response, as well as with greater remission and LDA, after 12 months of ABA treatment. However, previous studies have not found differences in effectiveness and safety between the use of ABA in monotherapy compared to ABA in combination with other DMARDs [51]. As for the clinical variables measuring the disease, the patients with lower baseline values for DAS28, NPJ, NSJ, PVAS, ESR, and HAQ had a better response to ABA treatment (greater EULAR response, LDA, and remission). These findings have been presented previously in other studies [52,53].

According to the results mentioned thus far, various biomarkers have been found as possible predictors of response to BTs in general and to ABA in particular. If we add to this the use of pharmacogenetics as a tool to achieve personalized medicine, treatments could be optimized so that the development of the disease could be delayed or arrested, avoiding the onset of irreversible disability [54]. The field of pharmacogenetics is one of those involved in this research process, as a number of SNPs have been implicated in the therapeutic response to various biological drugs [9,21,55]. Moreover, SNPs in the *FCGR2A* and *FCGR3A* genes have been studied as biomarkers of response to TNFis, RTX, or TCZ in various pathologies [4,9,11,22]. However, no study has investigated the involvement of these SNPs in the therapeutic response to ABA in patients with RA. Our results show that the *FCGR2A rs1801274* polymorphism is associated with the clinical effectiveness of ABA after 6 and 12 months of treatment. The *FCGR2A* rs1801274-AA genotype, considered to be of high affinity, has been associated with satisfactory EULAR response after 6 months and with LDA after 6 and 12 months of ABA treatment. In line with our results, a study conducted by Jiménez Morales et al. in 55 Caucasian patients (from Spain) treated with RTX showed that individuals carrying the *FCGR2A* rs1801274-AA genotype had a higher rate of remission after 6 months of treatment and greater EULAR response after 6, 12, and 18 months of treatment with RTX (*p* = 0.035; OR = 1.53, 95% CI = 1.11–21.12) [9]. However, in this same study, no association was found between the *FCGR2A* rs1801274 polymorphism and therapeutic response to TCZ in 98 Caucasian patients (from Spain) with RA [9]. Another study in 429 Caucasian patients (from Spain) diagnosed with RA and undergoing treatment with IFX showed that individuals carrying the *FCGR2A* rs1801274-G allele had a lower therapeutic response to this BT (*p* = 0.04) [56]. Avila-Pedretti et al. presented a study carried out in 348 Caucasian patients (from Spain) diagnosed with RA and treated with adalimumab (ADA), in which individuals carrying the *FCGR2A* rs1801274-GG genotype did not respond to ADA treatment after 12 weeks of treatment (GG vs. AG/AA; *p* = 0.022; OR = 2.54; 95% CI = 1.19–5.40) [46]. In contrast, the results of a meta-analysis performed in 3058 Caucasian patients diagnosed with RA undergoing TNFi therapy showed that Caucasian patients carrying the *FCGR2A* rs1801274-AA genotype had a lower EULAR response following treatment with ADA (*p* = 0.029; OR = 0.591, 95% CI = 0.37–0.95; I^2^ = 0, p_heterogeneity_ = 0.770) [22]. However, no significant results were obtained in patients treated with IFX and etanercept [22]. The association between the high-affinity FCGR2A-p.His131 variant and the better therapeutic response to ABA may be conditioned by an improved RA immune response [26]. Patients carrying the FCGR2A-p.His131 variant could present greater uptake and elimination of the autoimmune complexes produced by RA, decreasing tissue damage, and presenting a greater therapeutic response to ABA [26].

The *FCGR3A* rs396991 polymorphism has been investigated in numerous studies on RA and TNFi treatment [4,9,11,21,22]. No previous study has evaluated the association between the *FCGR3A* rs396991 polymorphism and ABA treatment in patients with RA. In our study, *FCGR3A* rs396991-A, a low-affinity allele, showed a tendency towards association with the EULAR response at 12 months of ABA therapy (A vs. CC; *p* = 0.078). Similarly, in a study conducted in 87 Caucasian patients (from Spain) diagnosed with RA, a significant association was found between the *FCGR3A* rs396991-AA genotype and greater EULAR response after 12 months of treatment with TCZ (AA vs. C; *p* = 0.027; OR = 5.08; 95% CI = 1.20–21.33) [9]. In this same study an association was found in 55 Caucasian patients (from Spain) diagnosed with RA and being treated with RTX between the *FCGR3A* rs396991-C allele and LDA after 18 months of treatment with RTX (C vs. AA; *p* = 0.077; OR = 4.90; 95% CI = 0.84-28.48) [9]. A meta-analysis of 1427 patients receiving TNFi, RTX, and IFX therapy showed that patients being treated with RTX who carried the *FCGR3A* rs396991-CC and *FCGR3A* rs396991-CA genotypes showed a lower therapeutic response (CC/CA vs. AA; *p* = 0.007; OR = 0.566; 95% CI = 0.37–0.86; I^2^ = 45.2, p_heterogeneity_ = 0.161) [57]. However, no association was found between the *FCGR3A* rs396991 polymorphism and effectiveness of TNFis (CC/CA vs. AA; *p* = 0.186) or IFX (CC/CA vs. AA; *p* = 0.065) [57]. Similarly, another meta-analysis performed by Montes et al. in 429 Caucasian patients diagnosed with RA and receiving TNFi therapy found no association between the *FCGR3A* rs396991 polymorphism and response to BTs (*p* = 0.5; OR = 1.11; 95% CI = 0.8–1.5; I^2^ = 62) [4]. The greater response to ABA in patients carrying the low affinity variant FCGR3A-p.158Phe could be due to the lower binding affinity of FCGR3A towards ABA so that the drug remains longer in the blood circulation, increasing the therapeutic response to ABA [4,9].

In our study, we analyzed the joint influence of the *FCGR2A* rs1801274-G (FCGR2A-p.131Arg) and *FCGR3A* rs396991-A (FCGR3A-p.158Phe) low-affinity alleles on the effectiveness of ABA in patients diagnosed with RA, since BTs, as we have already described, can be affected by both these SNPs. According to our results, an association was found between a larger number of low-affinity alleles and LDA after 12 months of treatment with ABA (Table S11). The presence of low-affinity FCGR2A/FCGR3A haplotypes could give rise to lower drug plasma clearance, increasing the half-life of ABA and therefore its effectiveness [14,21]. No study has evaluated the association between the additive power of the *FCGR2A* rs1801274-G and *FCGR3A* rs396991-A low-affinity alleles and ABA response in RA patients. A previous study in Asian patients (from Japan) diagnosed with RA and undergoing treatment with IFX showed that patients carrying low-affinity haplotypes had lower drug clearance compared to carriers of high-affinity haplotypes [14]. Furthermore, another study carried out by Dávila-Fajardo et al. in Caucasian patients (from Spain) treated with ADA showed an association between low-affinity haplotypes and satisfactory EULAR response (*p* = 0.017; OR = 1.53; 95% CI = 1.08–2.17) [21]. A study conducted in Caucasian patients (from Spain) with psoriatic arthritis treated with TNFi therapy found a significant association between low-affinity haplotypes and better therapeutic response after 6 to 8 weeks of treatment (*p* = 0.04) [58]. In our study, the presence of low-affinity *FCGR2A/FCGR3A* haplotypes was associated with greater clinical effectiveness of biological treatment; however, plasma ABA levels were not measured in our patients, and therefore we cannot correlate the pharmacogenetic response with the pharmacogenetics of ABA. These contradictory results regarding the influence of high- and low-affinity alleles in the *FCGR2A* rs1801274 and *FCGR3A* rs396991 SNPs could be determined by the different mechanisms of action of each of the BTs indicated for treatment of RA [4]. In addition, the differential expression of FCGR2A and FCGR3A in immune system cells could also influence the contradictory response results obtained between the two receptors [15]. A previous study demonstrated that ABA showed low affinity for the FCGR2 and FCGR3 receptors, and therefore its action through complement-dependent cytotoxicity and ADCC pathways is more limited than may be the case with other BTs that do act through these immunological pathways [9,59]. Furthermore, FCGR2A and FCGR3A effect on the pathophysiology of RA could also influence the response to therapies [26].

The limitation of our study was the sample size, which could be responsible for the loss of statistically significant association between the *FCGR2A* rs1801274 and *FCGR3A* rs396991 SNPs and remission after ABA treatment. Nevertheless, all the patients were recruited from the same hospital cohort following the same therapeutic protocols by the same team of rheumatologists, which ensured the homogeneity and reliability of the clinical variables collected. All the patients diagnosed during the study period were recruited, ensuring the representativeness of the sample. Despite the limited sample size, the effects observed in these patients were clear. Further studies will be required in larger cohorts to confirm the prognostic value of the *FCGR2A* rs1801274 and *FCGR3A* rs396991 polymorphisms and the response to ABA treatment in patients diagnosed with RA.

## 5. Conclusions

In conclusion, this study shows that patients with high-affinity FCGR2A-p.131His and low-affinity FCGR3A-p.158Phe could be associated with better therapeutic response to ABA in patients diagnosed with RA. The presence of low-affinity alleles of *FCGR2A* and *FCGR3A* was associated with greater clinical effectiveness and a lower rate of LDA after treatment with ABA. In addition, lower duration of previous BTs, the use of ABA in monotherapy, non-administration of concomitant GCs, greater duration of treatment with ABA, and early age of starting ABA therapy seem to be variables predictive of greater EULAR response, LDA, and remission in the individuals studied. As for clinical markers, lower baseline ESR and PVAS values are associated with better response to ABA therapy.

## Figures and Tables

**Table 1:** Clinical and demographic features of RA patients treated with abatacept.

Variables	Initial Level		
	N	(%)	Mean ± SD/p_50_(p_25_–p_75_)
Sex	120		
Women	89	74	–
**Smoking**			
Smoker	18	15	–
Ex-smoker	14	12	–
Non-smoker	88	73	–
**Age at Dx**	120	–	45.15 ± 13.72
**Years with RA**	120	–	24 (9–21)
**ABA start age**	120	–	56.63 ± 13.03
**ABA duration**	120	–	24.00 (14.75–44.25)
**Administration**			
SC	68	57	–
**Concomitant csDMARDs**			
**MTX**	42	35	–
**LFN**	14	12	–
**none**	64	53	–
**Concomitant GCs**			
Yes	102	85	–
**Monotherapy**			
No	113	94	–
**Number previous BTs**	120	–	2 (1–3)
**Duration previous BTs**	120	–	36 (24–72)
**Previous BTs**			
Naïve	15	12	–
1 TNF	31	26	–
2 TNFs	34	28	–
3 or more TNFs	40	33	–
**Reason for suspension**			
Primary failure	25	21	–
Secondary failure	12	10	–
AR	6	5	–
No suspension	77	64	–
**RF**			
Positive	96	80	–
**ACPA**			
Positive	85	71	–
**DAS28**	120	–	4.70 ± 1.43
**Baseline NPJ**	120	–	6 (3–10)
**Baseline NSJ**	120	–	3 (0–6)
**PVAS**	120	–	70 (50–80)
**Baseline CRP**	120	–	2 (1–4)
**Baseline ESR**	120	–	22 (10–38)
**HAQ**	120	–	1.75 (1.00–2.00)

ABA: abatacept; ACPA: anti-citrullinated protein antibody; AR: adverse reaction; BT: biological therapy; CRP: C-reactive protein; csDMARD: conventional synthetic disease-modifying antirheumatic drug; DAS28: disease activity score in 28 joints; Dx: diagnosis; ESR: erythrocyte sedimentation rate; GC: glucocorticoid; HAQ: Health Assessment Questionnaire; IV: intravenous; LFN: leflunomide; MTX: methotrexate; NPJ: number of painful joints: NSJ: number of swollen joints; p: percentile; PVAS: patient visual analogue scale; RA: rheumatoid arthritis; RF: rheumatoid factor; SC: subcutaneous; SD: standard deviation; TNFi: tumor necrosis factor inhibitor.

**Table 2:** Clinical Effectiveness of Abatacept in no-bionaïve and ABA-bionaïve patients.

No-Bionaïve Patients				
Response Variable	6 Months		12 Months	
	N	%	N	%
**EULAR response**	120		105	
Satisfactory	38	31.67	48	45.71
Unsatisfactory	82	68.33	57	54.29
**Remission (DAS28 < 2.6)**	18	15	29	27.62
**LDA (2.6** **≤ DAS28** **≤ 3.2)**	24	20	24	22.86
**ABA-bionaïve patients**				
**Response variable**	**6 months**		**12 months**	
**EULAR response**	15		14	
Satisfactory	8	53.33	11	78.57
Unsatisfactory	7	46.67	3	21.43
**Remission (DAS28 < 2.6)**	3	20	9	64.29
**LDA (2.6** **≤ DAS28** **≤ 3.2)**	5	33.33	3	21.43

DAS28: disease activity score in 28 joints; EULAR: European League Against Rheumatism criteria; LDA: low-activity disease; TNFi: tumor necrosis factor inhibitor.

**Table 3:** Predictors of response at 6 and 12 months of treatment with abatacept in rheumatoid arthritis patients (multivariate analysis).

Response Variable	Independent Variable	B	OR	*p*-Value (Variable)	95% CI	*R^2^*	Goodness of Fit
**6 MONTHS**							
**EULAR response**							
	Duration previous BTs	−0.017	0.98	0.006	0.97−0.99	Cox Snell *R^2^* = 0.173	χ^2^ = 9.750
	*FCGR2A* (AA vs. G)	0.887	2.43	0.048	1.01−5.92		
	Monotherapy (yes vs. no)	3.199	24.53	0.006	3.46−523.80	Nagelkerke *R^2^* = 0.243	*p* = 0.283
**LDA**							
	Initial PVAS	−0.033	0.97	0.003	0.95−0.99	Cox Snell *R^2^* = 0.110	χ^2^ = 9.606
	*FCGR2A* (AA vs. G)	1.149	3.16	0.022	1.19−8.66	Nagelkerke *R^2^* = 0.174	*p* = 0.294
**Remission**							
	ABA duration	0.023	1.02	0.026	1.01−1.04	Cox Snell *R^2^* = 0.232	χ^2^ = 3.338
	Duration previous BTs	−0.023	0.98	0.029	0.95−0.99		
	Initial ESR	−0.079	0.92	0.005	0.87−0.97	Nagelkerke *R^2^* = 0.406	*p* = 0.911
	Monotherapy (yes vs. no)	2.956	19.22	0.019	2.05−343.00		
**12 MONTHS**							
**EULAR response**							
	Initial PVAS	−0.056	0.95	<0.001	0.92−0.97	Cox Snell *R^2^* = 0.248	χ^2^ = 13.130
	Duration previous BTs	−0.012	0.99	0.029	0.98−0.99	Nagelkerke *R^2^* = 0.332	*p* = 0.108
**LDA**							
	ABA start age	0.059	1.06	0.007	1.02−1.11	Cox Snell *R^2^* = 0.196	χ^2^ = 15.030
	Concomitant GCs	−2.149	0.12	0.004	0.02−0.47		
	*FCGR2A* (AA vs. AG)	2.551	12.82	0.002	2.95−83.04	Nagelkerke *R^2^* = 0.297	*p* = 0.059
	*FCGR2A* (AA vs. GG)	1.890	6.62	0.036	1.25−46.89		
**Remission**							
	Duration previous BTs	−0.019	0.98	0.006	0.97–0.99	Cox Snell *R^2^* = 0.190	χ^2^ = 7.215
	Initial PVAS	−0.042	0.96	<0.001	0.94−0.98	Nagelkerke *R^2^* = 0.274	*p* = 0.514

ABA: abatacept; BT: biological therapy; CI: confidence interval; ESR: erythrocyte sedimentation rate; EULAR: European League Against Rheumatism criteria; GC: glucocorticoid; LDA: low disease activity; OR: odds ratio; PVAS: patient visual analogue scale.

**Table 4:** Haplotype association with EULAR response at 6 months of ABA adjusted by sex, duration of previous BTs, monotherapy, and initial DAS28.

FCGR2A	FCGR3A	Frequencies	Odds Ratio (95% CI)	*p*-Value
rs1801274	rs396991			
A	C	0.2746	1.00	–
G	A	0.2662	1.90 (0.66–5.49)	0.240
A	A	0.2629	0.90 (0.28–2.91)	0.860
G	C	0.1963	5.24 (0.98–28.08)	0.056

95% CI: confidence interval. Global haplotype association *p*-value: 0.013.

## Supplementary Materials

The following are available online at https://www.mdpi.com/article/10.3390/jpm11060573/s1, Table S1: Hardy-Weinberg equilibrium, Table S2: Linkage disequilibrium, Table S3: Minor allele frequencies of SNPs, Table S4: Haplotype frequency estimation EULAR response at 6 months of ABA, Table S5: Haplotype frequency estimation LDA at 6 months of ABA, Table S6: Haplotype frequency estimation remission at 6 months of ABA, Table S7: Haplotype frequency estimation EULAR response at 12 months of ABA, Table S8: Haplotype frequency estimation LDA at 12 months of ABA, Table S9: Haplotype frequency estimation remission at 12 months of ABA, Table S10: Predictors of EULAR response at 6 and 12 months of treatment with abatacept in rheumatoid arthritis patients (bivariate analysis), Table S11: Predictors of LDA at 6 and 12 months of treatment with abatacept in rheumatoid arthritis patients (bivariate analysis), Table S12: Predictors of remission at 6 and 12 months of treatment with abatacept in rheumatoid arthritis patients (bivariate analysis).

## Abbreviations

ABA	abatacept
ACPA	anti-cyclic citrullinated peptide antibodies
ACR	American College of Rheumatology
ADCC	antibody-dependent cellular cytotoxicity
Arg	arginine
bDMARDs	biologic disease-modifying antirheumatic drugs
BT	biological therapy
CRP	C-reactive protein
csDMARDs	conventional synthetic disease-modifying antirheumatic drugs
CTLA-4	cytotoxic T-lymphocyte-associated antigen 4
DAS28	28-joints Disease Activity Score
DMARDs	disease-modifying antirheumatic drugs
ESR	erythrocyte sedimentation rate
EULAR	European League Against Rheumatism
Fc	fragment crystallizable
FCGR	Fc-gamma receptor
GC	glucocorticoid
HAQ	Health Assessment Questionnaire score
His	histidine
HWE	Hardy–Weinberg equilibrium
IFX	infliximab
IgG1	human immunoglobulin G1
IV	intravenous
LDA	low-activity disease
LFN	leflunomide
MTX	methotrexate
NIJ	number of inflamed joints
NK	natural killer
NPJ	number of painful joints
OR	odds ratio
PCR	polymerase chain reaction
Phe	phenylalanine
PVAS	patient’s visual analogue scale
RA	rheumatoid arthritis
RF	rheumatoid factor
RTX	rituximab
SC	subcutaneous
SNP	single-nucleotide polymorphism
TCZ	tocilizumab
TNFi	tumor necrosis factor inhibitor
tsDMARDs	targeted synthetic disease-modifying antirheumatic drugs
Val	valine
