# Supplementary material for: Influence of the FCGR2A rs1801274 and FCGR3A rs396991 Polymorphisms on Response to Abatacept in Patients with Rheumatoid Arthritis
Source: J Pers Med. 2021 Jun 18;11(6):573. doi: 10.3390/jpm11060573 (PMC8233911; doi:10.3390/jpm11060573)
Supplement: Supplementary file 1 [file jpm-11-00573-s001.zip › Table S1. Hardy-Weinberg equilibrium.pdf]

Table S1. Hardy-Weinberg equilibrium

Chr	SNP	Sample	Minor Allele	Major Allele	Genotype counts	Observed heterozygosity	Expected heterozygosity	p-value
1	rs1801274	Cases	G	A	31/56/41	0.438	0.497	0.212
1	rs396991	Cases	C	A	23/72/33	0.563	0.497	0.159
Chr: chromosome; SNP: single-nucleotide polymorphism.								
