# Supplementary material for: Influence of the FCGR2A rs1801274 and FCGR3A rs396991 Polymorphisms on Response to Abatacept in Patients with Rheumatoid Arthritis
Source: J Pers Med. 2021 Jun 18;11(6):573. doi: 10.3390/jpm11060573 (PMC8233911; doi:10.3390/jpm11060573)
Supplement: Supplementary file 1 [file jpm-11-00573-s001.zip › Table S10. Predictors of EULAR response at 6 and 12 months of treatment with abatacept in rheumatoid arthritis patients (bivariate analysis).pdf]

Table S10. Predictors of EULAR response at 6 and 12 months of treatment with abatacept in rheumatoid arthritis patients (bivariate analysis)

	6 months							12 months					
Clinical variable	EULAR response						Clinical variable	EULAR response					
	N	Unsatisfactory	Satisfactory	p	OR	95% CI		N	Unsatisfactory	Satisfactory	p	OR	95% CI
**Sex**							**Sex**						
**Women**	89	63 (70.8)	26 (29.2)	0.373	–	–	**Women**	75	45 (60)	30 (40)	0.063	–	–
**Men**	31	19 (61.3)	12 (38.7)				**Men**	30	12 (40)	18 (60)			
**Smoking**							**Smoking**						
**Smoker**	18	12 (66.7)	6 (33.3)	0.602	–	–	**Smoker**	16	7 (43.8)	9 (56.2)	0.594	–	–
**Ex-smoker**	14	8 (57.1)	6 (42.9)				**Ex-smoker**	12	6 (50)	6 (50)			
**Non-smoker**	88	62 (70.5)	26 (29.5)				**Non-smoker**	77	44 (57.1)	33 (42.9)			
**Age at Dx**	120	45.22 ± 13.19	45.00 ± 14.97	0.935	–	–	**Age at Dx**	105	44.53 ± 13.61	46.44 ± 14.64	0.490	–	–
**Years with RA**	120	17 (12–22)	12 (7.25–18)	0.035	0.94	0.89 – 0.99	**Years with RA**	105	17 (12–23)	13.5 (8–18.5)	0.064	–	–
**ABA start age**	120	57.49 ± 12.87	54.76 ± 13.33	0.288	–	–	**ABA start age**	105	57.44 ± 13.40	56.48 ± 13.49	0.716	–	–
**ABA duration**	120	23 (13–35.75)	31.5 (16.25–48.75)	0.067	–	–	**ABA duration**	105	24 (16–44)	30.5 (20–50.5)	0.211	–	–
**Administr.**							**Administr.**						
**SC**	68	43 (63.2)	25 (36.8)	0.235	–	–	**SC**	60	31 (51.7)	29 (48.3)	0.534	–	–
**IV**	52	39 (75)	13 (25)				**IV**	45	26 (57.8)	19 (42.2)			
**Concomitant csDMARDs**							**Concomitant csDMARDs**						
**MTX**	42	30 (71.4)	12 (28.6)	0.592	–	–	**MTX**	37	20 (54.1)	17 (45.9)	0.998	–	–
**LFN**	14	8 (57.1)	6 (42.9)				**LFN**	13	7 (53.8)	6 (46.2)			
**none**	64	44 (68.8)	20 (31.2)				**none**	55	30 (54.5)	25 (45.5)			
**Concom. GCs**							**Concom. GCs**						

Yes	102	75 (73.5)	27 (26.5)	0.006	4.30	1.36–14.55	Yes	89	53 (59.6)	36 (40.4)	0.010	4.42	1.32–14.78
No	18	7 (38.9)	11 (61.1)				No	16	4 (25)	12 (75)			
**Monotherapy**							**Monotherapy**						
No	113	81 (71.7)	32 (28.3)	0.004	14.82	1.69–704.16	No	98	56 (57.1)	42 (42.9)	0.028	7.86	0.93–68.99
Yes	7	1 (14.3)	6 (85.7)				Yes	7	1 (14.3)	6 (85.7)			
**Number of previous BTs**	120	2 (1–3)	2(1–3)	0.106	–	–	**Number of previous BTs**	105	2 (1–3)	2 (1–3)	0.342	–	–
**Duration of previous BTs**	120	48 (24–84)	24 (9–45)	0.003	0.98	0.97–0.99	**Duration of previous BTs**	105	48 (24–84)	24 (9–51)	0.071	–	–
**Previous BTs**							**Previous BTs**						
Naive	15	7 (46.7)	8 (53.3)	0.271	–	–	Naive	14	3 (21.4)	11 (78.6)	0.028	0.17	0.03–0.71
1 TNFi	31	21 (67.7)	10 (32.3)				1 TNFi	28	17 (60.7)	11 (39.3)			
2 TNFis	34	25 (73.5)	9 (26.5)				2 TNFis	31	21 (67.7)	10 (32.3)			
3 or more TNFis	40	29 (72.5)	11 (27.5)				3 or more TNFis	32	16 (50)	16 (50)			
**RF**							**RF**						
Negative	24	16 (66.7)	8 (33.3)	0.844	–	–	Negative	22	11 (50)	11 (50)	0.649	–	–
Positive	96	66 (68.8)	30 (31.2)				Positive	83	46 (55.4)	37 (44.6)			
**ACPA**							**ACPA**						
Negative	35	28 (80)	7 (20)	0.088	–	–	Negative	30	16 (53.3)	14 (46.7)	0.901	–	–
Positive	85	54 (63.5)	31 (36.5)				Positive	75	41 (54.7)	34 (45.3)			
**DAS28**	120	5.14 ± 1.18	3.74 ± 1.47	<0.001	0.42	0.90 – 1.89	**DAS28**	105	4.95 ± 1.07	4.25 ± 1.63	0.013	0.68	0.49–0.91
**Baseline NPJ**	120	8 (4–11)	3 (0.25–6)	<0.001	0.84	0.75–0.92	**Baseline NPJ**	105	7 (4–10)	4.5 (2–8.25)	0.026	0.94	0.87–1.01
**Baseline NSJ**	120	3.5 (1–6)	1 (0–3)	<0.001	0.79	0.67–0.91	**Baseline NSJ**	105	3 (2–5)	1.5 (0–5)	0.103	–	–
**PVAS**	120	70 (60–80)	50 (22.5–60.0)	<0.001	0.94	0.92–0.97	**PVAS**	105	70 (60–80)	50 (30–70)	<0.001	0.95	0.93–0.97
**Baseline CRP**	120	2 (1–3.89)	2.55 (1.11–4.85)	0.394	–	–	**Baseline CRP**	105	2 (1–4.3)	2.04 (1.08–3.93)	0.992	–	–

**Baseline ESR**	120	27 (14–44.5)	14 (8–30)	0.010	0.97	0.94–0.99	**Baseline ESR**	105	22 (12–45)	21 (9.75–34.5)	0.387	–	–
**HAQ**	120	1.75 (1.50–2.00)	1.20 (0.75–1.73)	<0.001	0.32	0.16–0.58	**HAQ**	105	1.75 (1.25–2)	1.25 (0.75–2)	0.002	0.39	0.21–0.71
***FCGR2A* rs1801274**							***FCGR2A* rs1801274**						
**AA**	38	21 (55.3)	17 (44.7)	0.058	–	–	**AA**	35	22 (62.9)	13 (37.1)	0.352	–	–
**GG**	29	24 (82.8)	5 (17.2)				**GG**	25	14 (56)	11 (44)			
**AG**	53	37 (69.8)	16 (30.2)				**AG**	45	21 (46.7)	24 (53.3)			
**A**	91	58 (63.7)	33 (36.3)	0.067	–	–	**A**	80	43 (53.8)	37 (46.2)	0.844	–	–
**G**	82	61 (74.4)	21 (25.6)	0.056	–	–	**G**	70	35 (50)	35 (50)	0.299	–	–
***FCGR3A* rs396991**							***FCGR3A* rs396991**						
**CC**	22	17 (77.3)	5 (22.7)				**CC**	21	15 (71.4)	6 (28.6)			
**AA**	29	20 (69)	9 (31)	0.615	–	–	**AA**	23	10 (43.5)	13 (56.5)	0.162	–	–
**CA**	69	45 (65.2)	24 (34.8)				**CA**	61	32 (52.5)	29 (47.5)			
**C**	91	62 (68.1)	29 (31.9)	0.933	–	–	**C**	82	47 (57.3)	35 (42.7)	0.344	–	–
**A**	98	65 (66.3)	33 (33.7)	0.448	–	–	**A**	84	42 (50)	42 (50)	0.078	–	–
**Low-affinity allele *FCGR2A/FCGR3A***	120	2 (1–3)	2 (1–2)	0.155	–	–	**Low-affinity allele *FCGR2A/FCGR3A***	105	2 (1–2)	2 (1.75–3)	0.088	–	–

ABA: abatacept; ACPA: anti-citrullinated protein antibody; AR: adverse reaction; BT: biological therapy; CI: confidence interval; CRP: C-reactive protein; csDMARD: conventional synthetic disease-modifying antirheumatic drug; DAS28: disease activity score in 28 joints; Dx: diagnosis; ESR: erythrocyte sedimentation rate; EULAR: European League Against Rheumatism criteria; GC: glucocorticoid; HAQ: Health Assessment Questionnaire; IV: intravenous; LFN: leflunomide; MTX: methotrexate; NPJ: number of painful joints: NSJ: number of swollen joints; OR: odds ratio; PVAS: patient visual analogue scale; RA: rheumatoid arthritis; RF: rheumatoid factor; SC: subcutaneous; SD: standard deviation; TNFi: tumor necrosis factor inhibitor.
