# Supplementary material for: Influence of the FCGR2A rs1801274 and FCGR3A rs396991 Polymorphisms on Response to Abatacept in Patients with Rheumatoid Arthritis
Source: J Pers Med. 2021 Jun 18;11(6):573. doi: 10.3390/jpm11060573 (PMC8233911; doi:10.3390/jpm11060573)
Supplement: Supplementary file 1 [file jpm-11-00573-s001.zip › Table S11. Predictors of LDA at 6 and 12 months of treatment with abatacept in rheumatoid arthritis patients (bivariate analysis).pdf]

Table S11. Predictors of LDA at 6 and 12 months of treatment with abatacept in rheumatoid arthritis patients (bivariate analysis)

	6 MONTHS						12 MONTHS						
Clinical variable	Low disease activity						Clinical variable	Low disease activity					
	N	No LDA	LDA	p	OR	95% CI		N	No LDA	LDA	p	OR	95% CI
Sex							Sex						
Women	89	72 (80.9)	17 (19.1)	0.795	–	–	Women	75	60 (80)	15 (20)	0.308	–	–
Men	31	24 (77.4)	7 (22.6)				Men	30	21 (70)	9 (30)			
Smoking							Smoking						
Smoker	18	16 (88.9)	2 (11.1)	0.215	–	–	Smoker	16	11 (68.8)	5 (31.2)	0.135	–	–
Ex-smoker	14	9 (64.3)	5 (35.7)				Ex-smoker	12	7 (58.3)	5 (41.7)			
Non-smoker	88	71 (80.7)	17 (19.3)				Non-smoker	77	63 (81.8)	14 (18.2)			
Age at Dx	120	45.06±13.84	45.54±13.53	0.877	–	–	Age at Dx	105	44.11±14.09	49.75±13.31	0.084	–	–
Years with RA	120	16 (10–21)	13.5 (6.75–21.25)	0.239	–	–	Years with RA	105	14 (8–21)	16 (11.75–20.5)	0.494	–	–
ABA start age	120	56.83±13.13	55.79±12.86	0.728	–	–	ABA start age	105	55.54±13.68	61.92 ± 11.26	0.039	1.04	1.00–1.09
ABA duration	120	24 (14–45.25)	25.5 (15–39.50)	0.763	–	–	ABA duration	105	26 (17–46)	31 (23–66.25)	0.339	–	–
Administr.							Administr.						
SC	68	50 (73.5)	18 (26.5)	0.064	–	–	SC	60	48 (80)	12 (20)	0.485	–	–
IV	52	46 (88.5)	6 (11.5)				IV	45	33 (73.3)	12 (26.7)			
Concomitant csDMARDs							Concomitant csDMARDs						
MTX	42	36 (85.7)	6 (14.3)	0.509	–	–	MTX	37	28 (75.7)	9 (24.3)	0.788	–	–
LFN	14	11 (78.6)	3 (21.4)				LFN	13	11 (84.6)	2 (15.4)			

none	64	49 (76.6)	15 (23.4)				none	55	42 (76.4)	13 (23.6)			
**Concom. GCs**							**Concom. GCs**						
**Yes**	102	83 (81.4)	19 (18.6)	0.371	–	–	**Yes**	89	72 (80.9)	17 (19.1)	0.031	3.29	1.07–10.1
**No**	18	13 (72.2)	5 (27.8)				**No**	16	9 (56.2)	7 (43.8)			
**Monotherapy**							**Monotherapy**						
**No**	113	91 (80.5)	22 (19.5)	0.559	–	–	**No**	98	78 (79.6)	20 (20.4)	0.025	5.2	1.08–25.3
**Yes**	7	5 (71.4)	2 (28.6)				**Yes**	7	3 (42.9)	4 (57.1)			
**Number of previous BTs**	120	2 (1–3)	2 (1–3)	0.485	–	–	**Number of previous BTs**	105	2 (1–3)	2 (1–3)	0.665	–	–
**Duration of previous BTs**	120	48 (24–72)	24 (11–63)	0.064	–	–	**Duration of previous BTs**	105	24 (12–72)	42 (33–60.25)	0.336	–	–
**Previous BTs**							**Previous BTs**						
**Naive**	15	10 (66.7)	5 (33.3)	0.557	–	–	**Naive**	14	11 (78.6)	3 (21.4)	0.988	–	–
**1 TNFi**	31	26 (83.9)	5 (16.1)				**1 TNFi**	28	22 (78.6)	6 (21.4)			
**2 TNFis**	34	28 (82.4)	6 (17.6)				**2 TNFis**	31	24 (77.4)	7 (22.6)			
**3 or more TNFis**	40	32 (80)	8 (20)				**3 or more TNFis**	32	24 (75)	8 (25)			
**RF**							**RF**						
**Negative**	24	20 (83.3)	4 (16.7)	0.648	–	–	**Negative**	22	17 (77.3)	5 (22.7)	0.987	–	–
**Positive**	96	76 (79.2)	20 (20.8)				**Positive**	83	64 (77.1)	19 (22.9)			
**ACPA**							**ACPA**						
**Negative**	35	31 (88.6)	4 (11.4)	0.208	–	–	**Negative**	30	23 (76.7)	7 (23.3)	0.941	–	–
**Positive**	85	65 (76.5)	20 (23.5)				**Positive**	75	58 (77.3)	17 (22.7)			
**DAS28**	120	4.85 ± 1.42	4.07 ± 1.33	0.016	0.67	0.47–0.93	**DAS28**	105	4.69 ± 1.39	4.44 ± 1.39	0.448	–	–

**Baseline NPJ**	120	6.5 (3–10)	6 (0.75–8)	0.115	–	–	**Baseline NPJ**	105	6 (2–9)	5 (3–9.25)	0.881	–	–
**Baseline NSJ**	120	3 (0–6)	2 (0–3.25)	0.219	–	–	**Baseline NSJ**	105	3 (1–5)	2 (0–6)	0.862	–	–
**PVAS**	120	70 (50–80)	50 (30–60)	0.002	0.96	0.94–0.98	**PVAS**	105	70 (50–80)	60 (37.5–70)	0.191	–	–
**Baseline CRP**	120	2 (1–4)	2.65 (1.45–4.38)	0.409	–	–	**Baseline CRP**	105	2 (1.12–4.40)	2.05 (1–3.87)	0.619	–	–
**Baseline ESR**	120	24 (10–41.25)	20 (8–32.25)	0.439	–	–	**Baseline ESR**	105	22 (12–36)	15 (8.75–39.5)	0.569	–	–
**HAQ**	120	1.75 (1.18–2)	1.25 (0.79–2)	0.051	0.49	0.25–0.94	**HAQ**	105	1.75 (1.13–2)	1.44 (0.79–2)	0.362	–	–
***FCGR2A* rs1801274**							***FCGR2A* rs1801274**						
**AA**	38	26 (68.4)	12 (31.6)				**AA**	35	32 (91.4)	3 (8.6)			
**GG**	29	25 (86.2)	4 (13.8)	0.132	–	–	**GG**	25	19 (76)	6 (24)	0.029	5.33	1.57–24.70
**AG**	53	45 (84.9)	8 (15.1)				**AG**	45	30 (66.7)	15 (33.3)			
**A**	91	71 (78)	20 (22)	0.430	–	–	**A**	80	62 (77.8)	18 (22.5)	0.867	–	–
**G**	82	70 (85.4)	12 (14.6)	0.048	2.67	0.96–7.44	**G**	70	49 (70)	21 (30)	0.014	4.57	1.26–16.59
***FCGR3A* rs396991**							***FCGR3A* rs396991**						
**CC**	22	20 (90.9)	2 (9.1)				**CC**	21	17 (81)	4 (19)			
**AA**	29	22 (75.9)	7 (24.1)	0.354	–	–	**AA**	23	15 (65.2)	8 (34.8)	0.304	–	–
**CA**	69	54 (78.3)	15 (21.7)				**CA**	61	49 (80.3)	12 (19.7)			
**C**	91	74 (81.3)	17 (18.7)	0.595	–	–	**C**	82	66 (80.5)	16 (19.5)	0.123	–	–
**A**	98	76 (77.6)	22 (22.4)	0.157	–	–	**A**	84	64 (76.2)	20 (23.8)	0.642	–	–
**Low-affinity allele**<br>***FCGR2A/FCGR3A***	120	2 (1–3)	2 (1–2.25)	0.496	–	–	**Low-affinity allele**<br>***FCGR2A/FCGR3A***	105	2 (1–3)	2 (2–3)	0.044	1.59	1.01–2.58

ABA: abatacept; ACPA: anti-citrullinated protein antibody; AR: adverse reaction; BT: biological therapy; CI: confidence interval; CRP: C-reactive protein; csDMARD: conventional synthetic disease-modifying antirheumatic drug; DAS28: disease activity score in 28 joints; Dx: diagnosis; ESR: erythrocyte sedimentation rate; EULAR: European League Against Rheumatism criteria; GC: glucocorticoid; HAQ: Health Assessment Questionnaire; IV: intravenous; LFN: leflunomide; MTX: methotrexate; NPJ: number of painful joints: NSJ: number of swollen joints; OR: odds ratio; PVAS: patient visual analogue scale; RA: rheumatoid arthritis; RF: rheumatoid factor; SC: subcutaneous; SD: standard deviation; TNFi: tumor necrosis factor inhibitor.
