# Supplementary material for: Influence of the FCGR2A rs1801274 and FCGR3A rs396991 Polymorphisms on Response to Abatacept in Patients with Rheumatoid Arthritis
Source: J Pers Med. 2021 Jun 18;11(6):573. doi: 10.3390/jpm11060573 (PMC8233911; doi:10.3390/jpm11060573)
Supplement: Supplementary file 1 [file jpm-11-00573-s001.zip › Table S12. Predictors of remission at 6 and 12 months of treatment with abatacept in rheumatoid arthritis patients (bivariate analysis) Bivariante remision_nuevo_EN.pdf]

Table S12. Predictors of remission at 6 and 12 months of treatment with abatacept in rheumatoid arthritis patients (bivariate analysis)

	6 MONTHS							12 MONTHS					
**Clinical variable**	**Remission**						**Clinical variable**	**Remission**					
	**N**	**No Remission**	**Remission**	**p**	**OR**	**95% CI**		**N**	**No Remission**	**Remission**	**p**	**OR**	**95% CI**
**Sex**							**Sex**						
**Women**	89	78 (87.6)	11 (12.4)	0.169	–	–	**Women**	75	56 (74.7)	19 (25.3)	0.471	–	–
**Men**	31	24 (77.4)	7 (22.6)				**Men**	30	20 (66.7)	10 (33.3)			
**Smoking**							**Smoking**						
**Smoker**	18	13 (72.2)	5 (27.8)	0.211	–	–	**Smoker**	16	11 (68.8)	5 (31.2)	0.816	–	–
**Ex-smoker**	14	13 (92.9)	1 (7.1)				**Ex-smoker**	12	8 (66.7)	4 (33.3)			
**Non-smoker**	88	76 (86.4)	12 (13.6)				**Non-smoker**	77	57 (74)	20 (26)			
**Age at Dx**	120	45.56 ± 13.84	42.83 ± 13.53	0.439	–	–	**Age at Dx**	105	45.76 ± 13.64	44.44 ± 15.29	0.670	–	–
**Years with RA**	120	16 (10–21)	13.5 (6.75–21.25)	0.142	–	–	**Years with RA**	105	16.5 (12–22.25)	10 (7–18)	0.655	–	–
**ABA start age**	120	57.52 ± 13.13	51.56 ± 12.86	0.073	–	–	**ABA start age**	105	58.46 ± 13.03	53.17 ± 13.77	0.070	–	–
**ABA duration**	120	24 (14–45.25)	25.5 (15–39.50)	0.015	1.02	1.01–1.03	**ABA duration**	105	25 (16–42.5)	33 (22–55)	0.087	–	–
**Administr.**							**Administr.**						
**SC**	68	59 (86.8)	9 (13.2)	0.609	–	–	**SC**	60	41 (68.3)	19 (31.7)	0.378	–	–
**IV**	52	43 (82.7)	9 (17.3)				**IV**	45	35 (77.8)	10 (22.2)			
**Concomitant csDMARDs**							**Concomitant csDMARDs**						
**MTX**	42	36 (85.7)	6 (14.3)	0.308	–	–	**MTX**	37	25 (67.6)	12 (32.4)	0.628	–	–

**LFN**	14	10 (71.4)	4 (28.6)				**LFN**	13	9 (69.2)	4 (30.8)			
**none**	64	56 (87.5)	8 (12.5)				**none**	55	42 (76.4)	13 (23.6)			
**Concom. GCs**							**Concom. GCs**						
**Yes**	102	91 (89.2)	11 (10.8)	0.002	5.16	1.4–18.58	**Yes**	89	66 (74.2)	23 (25.8)	0.337	–	–
**No**	18	11 (61.1)	7 (38.9)				**No**	16	10 (62.5)	6 (37.5)			
**Monotherapy**							**Monotherapy**						
**No**	113	99 (87.6)	14 (12.4)	0.001	9.13	1.39–68.97	**No**	98	71 (72.4)	27 (27.6)	0.954	–	–
**Yes**	7	3 (42.9)	4 (57.1)				**Yes**	7	5 (71.4)	2 (28.6)			
**Number of previous BTs**	120	2 (1–3)	2 (1–3)	0.212	–	–	**Number of previous BTs**	105	2 (1–3)	1 (0–2)	0.038	0.64	0.42–0.94
**Duration of previous BTs**	120	48 (24–72)	24 (11–63)	0.041	0.98	0.96–0.99	**Duration of previous BTs**	105	48 (24–84)	24 (0–36)	0.002	0.98	0.97–0.99
**Previous BTs**							**Previous BTs**						
**Naive**	15	12 (80)	3 (20)	0.672	–	–	**Naive**	14	5 (35.7)	9 (64.3)	0.012	6.60	1.67–29.62
**1 TNFi**	31	25 (80.6)	6 (19.4)				**1 TNFi**	28	22 (78.6)	6 (21.4)			
**2 TNFis**	34	29 (85.3)	5 (14.7)				**2 TNFis**	31	24 (77.4)	7 (22.6)			
**3 or more TNFis**	40	36 (90)	4 (10)				**3 or more TNFis**	32	25 (78.1)	7 (21.9)			
**RF**							**RF**						
**Negative**	24	20 (83.3)	4 (16.7)	0.798	–	–	**Negative**	22	15 (68.2)	7 (31.8)	0.603	–	–
**Positive**	96	82 (85.4)	14 (14.6)				**Positive**	83	61 (73.5)	22 (26.5)			
**ACPA**							**ACPA**						

**Negative**	35	31 (88.6)	4 (11.4)	0.583	–	–	**Negative**	30	22 (73.3)	8 (26.7)	0.892	–	–
**Positive**	85	71 (83.5)	14 (16.5)				**Positive**	75	54 (72)	21 (28)			
**DAS28**	120	4.92 ± 1.42	3.43 ± 1.33	<0.001	0.44	0.27–0.66	**DAS28**	105	4.79 ± 1.23	4.20 ± 1.71	0.097	–	–
**Baseline NPJ**	120	6.5 (3–10)	6 (0.75–8)	<0.001	0.79	0.66–0.91	**Baseline NPJ**	105	7 (3–10)	5 (2–8)	0.027	0.91	0.82–1.00
**Baseline NSJ**	120	3 (0–6)	2 (0–3.25)	<0.001	0.68	0.49–0.86	**Baseline NSJ**	105	3 (1–6)	1 (0–4)	0.112	–	–
**PVAS**	120	70 (50–80)	50 (30–60)	<0.001	0.95	0.92–0.97	**PVAS**	105	70 (57.5–80)	50 (30–70)	<0.001	0.97	0.95–0.99
**Baseline CRP**	120	2 (1–4)	2.65 (1.45 –4.38)	0.738	–	–	**Baseline CRP**	105	2 (1–4)	2 (1.1–4.4)	0.946	–	–
**Baseline ESR**	120	24 (10–41.25)	20 (8–32.25)	<0.001	0.94	0.89–0.98	**Baseline ESR**	105	21.5 (10–44.25)	22 (11–32)	0.561	–	–
**HAQ**	120	1.75 (1.18–2)	1.25 (0.79–2)	0.001	0.32	0.14–0.67	**HAQ**	105	1.75 (1.20–2.00)	1.20 (0.75–1.60)	0.006	0.43	0.22–0.80
***FCGR2A* rs1801274**							***FCGR2A* rs1801274**						
**AA**	38	33 (86.8)	5 (13.2)	0.226	–	–	**AA**	35	25 (71.4)	10 (28.6)	0.981	–	–
**GG**	29	27 (93.1)	2 (6.9)				**GG**	25	18 (72)	7 (28)			
**AG**	53	42 (79.2)	11 (20.8)				**AG**	45	33 (73.3)	12 (26.7)			
**A**	91	75 (82.4)	16 (17.6)	0.161	–	–	**A**	80	58 (72.5)	22 (27.5)	0.961	–	–
**G**	82	69 (84.1)	13 (15.9)	0.789	–	–	**G**	70	51 (72.9)	19 (27.1)	0.877	–	–
***FCGR3A* rs396991**							***FCGR3A* rs396991**						
**CC**	22	19 (86.4)	3 (13.6)	0.313	–	–	**CC**	21	18 (85.7)	3 (14.3)	0.311	–	–
**AA**	29	27 (93.1)	2 (6.9)				**AA**	23	16 (69.6)	7 (30.4)			
**CA**	69	56 (81.2)	13 (18.8)				**CA**	61	42 (68.9)	19 (31.1)			
**C**	91	75 (82.4)	16 (17.6)	0.161	–	–	**C**	82	60 (73.2)	22 (26.8)	0.794	–	–
**A**	98	83 (84.7)	15 (15.3)	0.843	–	–	**A**	84	58 (69)	26 (31)	0.175	–	–

**Low-affinity allele** ***FCGR2A/FCGR3A***	120	2 (1–3)	2 (1.25–2)	0.438	–	–	**Low-affinity allele** ***FCGR2A/FCGR3A***	105	2 (1–2)	2 (1–3)	0.406	–	–

ABA: abatacept; ACPA: anti-citrullinated protein antibody; AR: adverse reaction; BT: biological therapy; CI: confidence interval; CRP: C-reactive protein; csDMARD: conventional synthetic disease-modifying antirheumatic drug; DAS28: disease activity score in 28 joints; Dx: diagnosis; ESR: erythrocyte sedimentation rate; EULAR: European League Against Rheumatism criteria; GC: glucocorticoid; HAQ: Health Assessment Questionnaire; IV: intravenous; LFN: leflunomide; MTX: methotrexate; NPJ: number of painful joints: NSJ: number of swollen joints; OR: odds ratio; PVAS: patient visual analogue scale; RA: rheumatoid arthritis; RF: rheumatoid factor; SC: subcutaneous; SD: standard deviation; TNFi: tumor necrosis factor inhibitor.
