# Supplementary material for: Influence of the FCGR2A rs1801274 and FCGR3A rs396991 Polymorphisms on Response to Abatacept in Patients with Rheumatoid Arthritis
Source: J Pers Med. 2021 Jun 18;11(6):573. doi: 10.3390/jpm11060573 (PMC8233911; doi:10.3390/jpm11060573)
Supplement: Supplementary file 1 [file jpm-11-00573-s001.zip › Table S2. Linkage disequilibrium.pdf]

Table S2. Linkage disequilibrium

Chr	BP	SNP	Chr	BP	SNP	R^2	D'
1	161509955	rs1801274	1	161544752	rs396991	0.009	0.110

Chr: chromosome; BP: physical position (base-pair); SNP: single-nucleotide polymorphism.
