# Supplementary material for: Influence of the FCGR2A rs1801274 and FCGR3A rs396991 Polymorphisms on Response to Abatacept in Patients with Rheumatoid Arthritis
Source: J Pers Med. 2021 Jun 18;11(6):573. doi: 10.3390/jpm11060573 (PMC8233911; doi:10.3390/jpm11060573)
Supplement: Supplementary file 1 [file jpm-11-00573-s001.zip › Table S3. Minor allele frequencies of SNPs.pdf]

Table S3. Minor allele frequencies of SNPs.

Chr	SNP	Minor allele	Major alelle	MAF
1	rs1801274	G	A	0.4609
1	rs396991	C	A	0.4609
Chr: chromosome; MAF: minor allele frequency: SNP: single-nucleotide polymorphism.				
