# Supplementary material for: Influence of the FCGR2A rs1801274 and FCGR3A rs396991 Polymorphisms on Response to Abatacept in Patients with Rheumatoid Arthritis
Source: J Pers Med. 2021 Jun 18;11(6):573. doi: 10.3390/jpm11060573 (PMC8233911; doi:10.3390/jpm11060573)
Supplement: Supplementary file 1 [file jpm-11-00573-s001.zip › Table S5. Haplotype frequency estimation LDA at 6 months of ABA.pdf]

Table S5. Haplotype frequency estimation: LDA at 6 months of ABA

FCGR2A rs1801274	***FCGR3A*** rs396991	**Total**	**LDA**	**No LDA**	**Cumulative frequency**
A	C	0.2733	0.3281	0.2644	0.2733
G	A	0.265	0.2656	0.2696	0.5383
A	A	0.2642	0.3385	0.2408	0.8025
G	C	0.1975	0.0677	0.2252	1
