# Supplementary material for: Influence of the FCGR2A rs1801274 and FCGR3A rs396991 Polymorphisms on Response to Abatacept in Patients with Rheumatoid Arthritis
Source: J Pers Med. 2021 Jun 18;11(6):573. doi: 10.3390/jpm11060573 (PMC8233911; doi:10.3390/jpm11060573)
Supplement: Supplementary file 1 [file jpm-11-00573-s001.zip › Table S7. Haplotype frequency estimation EULAR response at 12 months of ABA.pdf]

Table S7. Haplotype frequency estimation: EULAR response at 12 months of ABA

FCGR2A rs1801274	***FCGR3A*** rs396991	**Total**	**EULAR response satisfactory**	**EULAR response unsatisfactory**	**Cumulative frequency**
A	C	0.2909	0.2267	0.3388	0.2909
A	A	0.2567	0.2941	0.2314	0.5476
G	A	0.2528	0.2788	0.2248	0.8004
G	C	0.1996	0.2004	0.205	1
