# Supplementary material for: Influence of the FCGR2A rs1801274 and FCGR3A rs396991 Polymorphisms on Response to Abatacept in Patients with Rheumatoid Arthritis
Source: J Pers Med. 2021 Jun 18;11(6):573. doi: 10.3390/jpm11060573 (PMC8233911; doi:10.3390/jpm11060573)
Supplement: Supplementary file 1 [file jpm-11-00573-s001.zip › Table S8. Haplotype frequency estimation LDA at 12 months of ABA.pdf]

Table S8. Haplotype frequency estimation: LDA at 12 months of ABA

FCGR2A rs1801274	***FCGR3A*** rs396991	**Total**	**LDA**	**No LDA**	**Cumulative frequency**
A	C	0.2909	0.0945	0.3321	0.2909
A	A	0.2567	0.343	0.2481	0.5476
G	A	0.2528	0.2403	0.2395	0.8004
G	C	0.1996	0.3222	0.1802	1
